# Preoperative aspirin use and acute kidney injury after cardiac surgery: A propensity-score matched observational study

**DOI:** 10.1371/journal.pone.0177201

**Published:** 2017-05-04

**Authors:** Min Hur, Chang-Hoon Koo, Hyung-Chul Lee, Sun-Kyung Park, Minkyung Kim, Won Ho Kim, Jin-Tae Kim, Jae-Hyon Bahk

**Affiliations:** 1 Department of Anesthesiology and Pain Medicine, Seoul National University Hospital, Seoul, Republic of Korea; 2 Department of Anesthesiology and Pain Medicine, CHA Bundang Medical Center, CHA University, Seongnam-si, Kyeonggi-do, Republic of Korea; Universita degli Studi di Bologna, ITALY

## Abstract

**Background:**

The association between preoperative aspirin use and postoperative acute kidney injury (AKI) in cardiovascular surgery is unclear. We sought to evaluate the effect of preoperative aspirin use on postoperative AKI in cardiac surgery.

**Methods:**

A total of 770 patients who underwent cardiovascular surgery under cardiopulmonary bypass were reviewed. Perioperative clinical parameters including preoperative aspirin administration were retrieved. We matched 108 patients who took preoperative aspirin continuously with patients who stopped aspirin more than 7 days or did not take aspirin for the month before surgery. The parameters used in the matching included variables related to surgery type, patient’s demographics, underlying medical conditions and preoperative medications.

**Results:**

In the first seven postoperative days, 399 patients (51.8%) developed AKI, as defined by the Kidney Disease Improving Global Outcomes (KDIGO) criteria and 128 patients (16.6%) required hemodialysis. Most patients took aspirin 100 mg once daily (n = 195, 96.5%) and the remaining 75 mg once daily. Multivariable analysis showed that preoperative maintenance of aspirin was independently associated with decreased incidence of postoperative AKI (odds ratio [OR] 0.46, 95% confidence interval [CI] 0.21–0.98, *P* = 0.048; after propensity score matching: OR 0.39, 95% CI 0.22–0.67, *P* = 0.001). Preoperative maintenance of aspirin was associated with less incidence of AKI defined by KDIGO both in the entire and matched cohort (n = 44 [40.7%] vs. 69 [63.9%] in aspirin and non-aspirin group, respectively in matched sample, relative risk [RR] 0.64, 95% CI 0.49, 0.83, *P* = 0.001). Preoperative aspirin was associated with decreased postoperative hospital stay after matching (12 [9–18] days vs. 16 [10–25] in aspirin and non-aspirin group, respectively, *P* = 0.038). Intraoperative estimated or calculated blood loss using hematocrit difference and estimated total blood volume showed no difference according to aspirin administration in both entire and matched cohort.

**Conclusions:**

Preoperative low dose aspirin administration without discontinuation was protective against postoperative AKI defined by KDIGO criteria independently in both entire and matched cohort. Preoperative aspirin was also associated with decreased hemodialysis requirements and decreased postoperative hospital stay without increasing bleeding. However, differences in AKI and hospital stay were not associated with in-hospital mortality.

## Introduction

Aspirin has both anti-inflammatory and antiplatelet effect and has been regarded as an essential medication to prevent cardiovascular disease. Literatures reported that aspirin decreases the incidence of myocardial infarction, stroke and all-cause mortality [1,2]. American Heart Association (AHA) guidelines updated in 2011 recommended that high-risk patients with coronary artery disease, cerebrovascular disease, and peripheral vascular disease should be prescribed aspirin indefinitely if the risk of bleeding did not outweigh the benefit [3].

However, the association between preoperative aspirin and the outcomes of cardiac surgery were relatively rare and the results so far were not consistent [4–6]. Previous observational studies have reported that aspirin administration prior to cardiac surgery was associated with decreased postoperative cardiovascular and cerebral complications, renal failure, length of hospital stay and short-term mortality without significant increase in bleeding risk [7–12]. However, there are also studies reporting no difference in the postoperative composite outcomes and increased bleeding complications [13–15].

Acute kidney injury (AKI) is an important complication after major cardiac and aortic surgery with its incidence up to 55% and was reported to be associated with increased mortality [16]. The etiology of cardiac-surgery associated AKI was reported to be multifactorial, including hemodynamic derangement, renal ischemia-reperfusion injury, inflammation and oxidative stress [17,18]. Surgical stress is considered to be thrombogenic and may result in impaired microvascular circulation and thereby renal ischemia. If the effect of antiplatelet agent on the surgical bleeding is not greater than the effect on the renal microvascular circulation, the administration of antiplatelet agent prior to surgery can be protective against AKI after surgery or vice versa. Also anti-inflammatory action of aspirin may mitigate the inflammatory process that may play a major role in the pathogenesis of AKI resulting from ischemia [19,20]. However, the association between preoperative aspirin and postoperative AKI has not been evaluated fully, although some studies reported outcome of renal failure according to aspirin discontinuation [9].

Therefore, the authors attempt to test a hypothesis that preoperative aspirin administration before major cardiac surgery may be associated with decreased incidence of postoperative AKI. We also evaluate the effect of preoperative aspirin administration with postoperative clinical outcomes including short-term mortality and major morbidity. To evaluate the association between preoperative aspirin administration, surgical bleeding and postoperative clinical outcomes including AKI, we conducted a retrospective observational study and sought to compare the patients with preoperative aspirin without discontinuation and those without preoperative aspirin by propensity-score matched retrospective case-control study.

## Materials and methods

**Fig 1:**
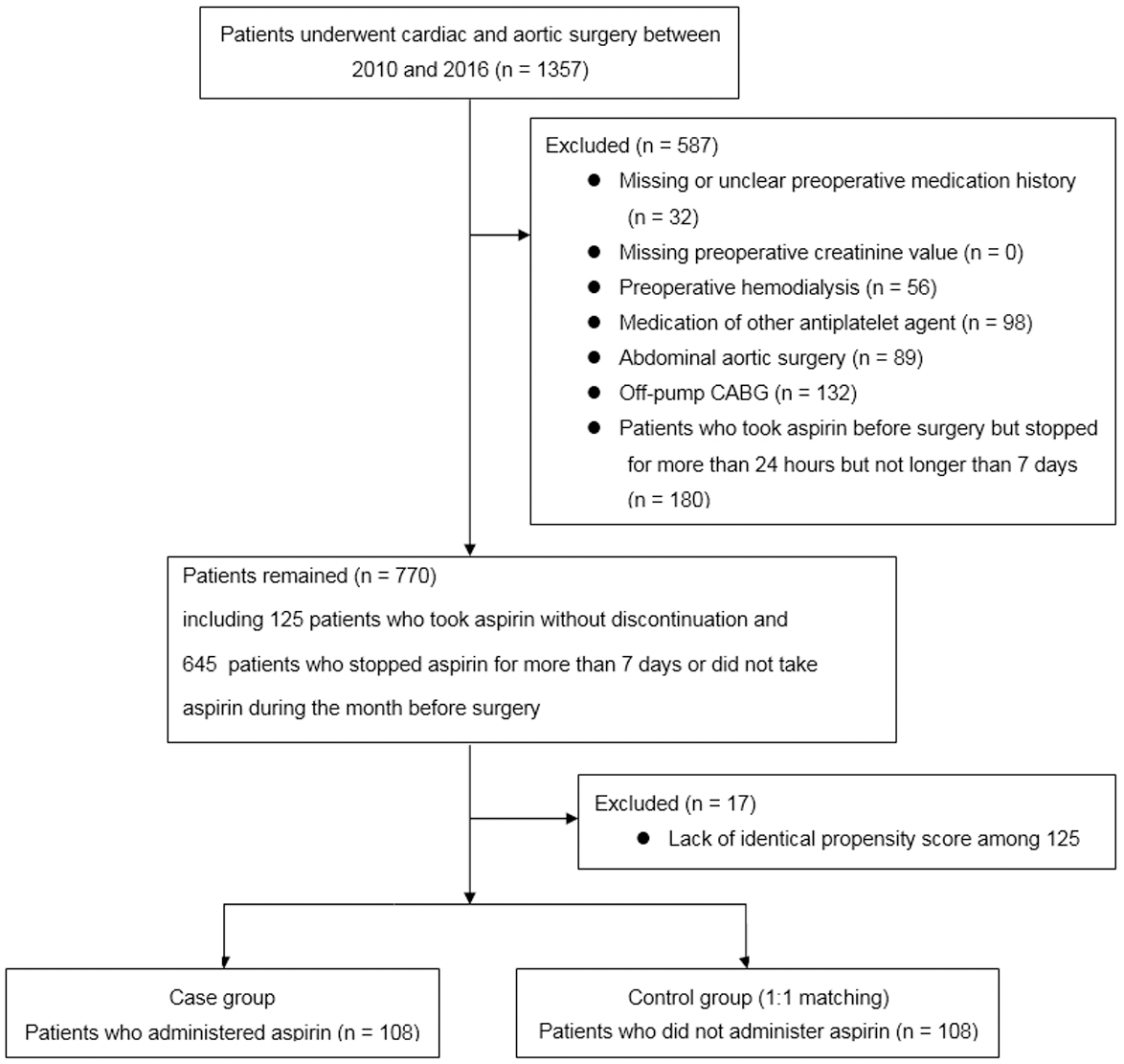
Flow diagram outlining the inclusion and exclusion criteria and study design. CABG = coronary artery bypass graft.

This study was approved from the Seoul National University Institutional Review Board (1608-126-788), and the requirement for patient informed consent was waived, given the retrospective design of the study. The electronic medical records were retrospectively reviewed in 1357 consecutive adult patients who had undergone elective cardiac and aortic surgery under cardiopulmonary bypass (CPB) at the reporting single institution between January 2010 and May 2016 (Fig 1). Surgery included coronary artery bypass graft (CABG), valvular heart surgery, thoracic and abdominal aortic surgery, cardiac myxoma resection and adult atrial or ventricular septal defect closure. Exclusion criteria included the followings: if they had missing or unclear preoperative medication history (n = 32), missing preoperative creatinine value (n = 0), preoperative hemodialysis (n = 56) and medication of glycoprotein IIb/IIIa antagonists including abciximab, eptifibatide, and tirofiban or adenosine diphosphate receptor inhibitor including clopidogrel, prasugrel, ticlopidine or ticagrelor (n = 98). Then, patients who underwent abdominal aortic surgery (n = 89) and off-pump CABG (n = 132) were excluded because these patients did not underwent CPB. The remaining 950 patients were reviewed. Those patients who took aspirin before surgery but stopped for more than 24 hours but not longer than 7 days (n = 180) were excluded from the final logistic regression analysis because these patients may have residual effect of aspirin and this may act as bias. The analysis of 950 patients including the patients with possible residual aspirin effect (n = 180) was shown in S1 Table.

### Perioperative clinical variables and antiplatelet agent administration

Baseline perioperative clinical parameters previously demonstrated to be associated with postoperative AKI were obtained in this study [21–29]. They included patient history, medication history, preoperative cardiac history and systolic function, anesthesia and surgery-associated factors, laboratory findings, intraoperative hemodynamic variables, and transfusion amounts.

We investigated patient’s history of preoperative medication of all kind including antiplatelet agents and whether antiplatelet agents were stopped before surgery. Duration of discontinuation was obtained by carefully reviewing preoperative physician’s daily order. Patients were classified into three groups of aspirin medication pattern according to their maintenance of aspirin before surgery. If a patient who maintained aspirin before surgery for more than 7 days and administered aspirin within 24 hours of surgery, the patient was classified as aspirin group. If a patient who administered aspirin before surgery but stopped for more than 24 hours but not more than 7 days before surgery, the patient was designated as possible residual aspirin effect group, or a patient stop aspirin for more than 7 days before surgery or did not take aspirin within 30 days before surgery, the patient was classified as no-aspirin group. If patient took any of the antiplatelet agents other than aspirin listed in the exclusion criteria within five days before surgery, the patient were excluded.

### Outcome variable and associated comorbidities

The development of AKI within the first postoperative seven days was the primary measured outcome. We defined AKI according to the Kidney Disease Improving Global Outcomes (KDIGO) criteria [30], which classifies AKI based on the maximal increase in sCr from preoperative baseline levels. KDIGO stages were determined as the highest possible stage according to creatinine or urine output criteria within postoperative 7 days [30,31]. All patients who met the KDIGO criteria for stage one, two, and three were considered to have developed AKI. Intraoperative total bleeding amounts and transfusion requirements recorded in the anesthesia record were obtained. Bleeding amounts during the days of surgery was calculated according to the difference between preoperative hematocrit and postoperative day one nadir hematocrit by the following equation [32].

$$\text{Calculated bleeding amount during the day of surgery}=3\times \text{ Estimated total blood volume}\times \left(\frac{\text{Preoperative hematocrit}\left(\text{\%}\right)}{100}-\frac{\text{nadir hematocrit during the day of surgery}\left(\text{\%}\right)}{100}\right)$$

Postoperative outcome variables included the need for new-onset hemodialysis after surgery, intra-aortic balloon pump (IABP) insertion, requirement for extracorporeal membrane oxygenation (ECMO) insertion, postoperative new-onset atrial fibrillation, length of hospital and ICU stay, and in-hospital all-cause mortality. We compared the incidence of postoperative complications including major adverse cardiovascular event (MACE), new-onset atrial fibrillation and resternotomy due to postoperative bleeding between aspirin and no-aspirin groups. MACE was defined as a composite of non-fatal myocardial infarction, coronary revascularization, pulmonary embolism and stroke. Intraoperative bleeding amount was also evaluated as a secondary outcome. Postoperative incidence of AKI was also investigated in terms of acute kidney injury network criteria (AKIN) determined during the postoperative two days [30].

### Statistical analysis

All statistical analyses were performed using the SPSS software (version 22.0, IBM Corp., Armonk, NY, USA). Propensity score matching was conducted by Propensity Score Matching for SPSS (version 3.0.4., Cornell University). For all analyses, *P* <0.05 was considered statistically significant. A sample size of 400 patients or more was suggested under the assumption that the expected odds ratio (OR) of AKI development in patients who stopped aspirin before surgery > 24 hours would be 2.0, with a type I error of 0.05 and a power of 0.8.[33] We also validated the sample size according to a rule that the number of outcome events should be ten per every independent predictor [34]. For the present study, this was calculated to be 500 patients or more to allow unbiased accommodation of less than ten predictors in a multivariable logistic regression analysis under the assumption of at least 20% incidence of postoperative AKI [34].

Median [interquartile range] was used for continuous parameters and a number (frequency, %) was used for categorical parameters. Missing data other than preoperative sCr was present in less than 2% of records. We substituted the most frequent gender-specific value for missing values of the categorical variables and gender-specific median values for missing values of the continuous variables before propensity-score matching. Fisher’s exact test or the chi-square test was used for comparison of categorical variables according to their expected counts. The two sample *t*-test or the Mann-Whitney *U* test was used to compare continuous variables between those with and without AKI according to their distribution. Logistic regression analyses were used to identify risk factors for AKI. Possible risk factors for postoperative AKI was identified by univariable logistic regression analysis. Then, multivariable regression analysis was performed including only variables that were significant on univariable analyses (*P* <0.05) by stepwise backward variable selection. Variables that have commonly used normal cutoffs, including albumin, uric acid, serum creatinine and mean arterial pressure were categorized using their normal cut-offs. Independent predictors were selected from a list of potential predictors of univariable analysis by performing both forward and backward stepwise variable elimination process with a significance criterion of *P*<0.05.

A propensity score analysis was used to match patients with and without AKI, to reduce any potential selection bias from demographic factors and underlying medical conditions and medication history. Binary logistic regression analysis, including interaction terms, was used to determine the probability of AKI and non-AKI group assignment, which was used for matching. Patients were matched using a greedy method with 1:1 pair. A total of 108 patients with preoperative aspirin were matched with those who did not administer aspirin within 24 hours before surgery using the nearest neighbor matching. All the variables listed in Table 1 as well as operation and CPB time were used as contributors to the propensity score: demographic data, medical history, variables related to cardiac status, surgery type and preoperative medications other than aspirin. We defined the caliper as 0.2 standard deviation of the logit-transformed propensity score. The balance between the two groups was tested by paired comparison of contributor variables. Multivariable logistic regression analysis was again performed in the matched sample.

**Table 1:** Baseline patient characteristics in the entire and matched cohort.

Characteristic	Entire cohort			Matched cohort		
	Aspirin group (n = 125)	Non-Aspirin group (n = 645)	*P*-value	Aspirin group (n = 108)	Non-Aspirin group (n = 108)	*P*-value
**Demographic data**						
**Age, years**	72 [66–78]	67 [60–74]	<0.001	72 [64–72]	69 [62–77]	0.378
**Female, n**	44 (35.2)	334 (51.8)	0.001	42 (38.9)	44 (40.7)	0.781
**Body-mass index, kg/m**^**2**^	23.1 [20.6–25.1]	22.3 [20.0–24.5]	0.036	23.1 [21.0–25.1]	22.7 [20.8–24.9]	0.498
**Medical history**						
**Hypertension, n**	62 (49.6)	260 (40.3)	0.054	54 (50.0)	53 (49.1)	0.892
**Diabetes mellitus, n**	32 (25.6)	90 (14.0)	0.001	24 (22.2)	21 (19.4)	0.615
**Stroke, n**	3 (2.4)	17 (2.6)	0.999	3 (2.8)	3 (2.8)	0.999
**Chronic kidney disease, n**	11 (8.8)	29 (4.5)	0.047	9 (8.3)	8 (7.4)	0.801
**Angina pectoris, n**	34 (27.2)	24 (3.7)	<0.001	21 (19.4)	19 (17.6)	0.726
**Myocardial infarction, n**	15 (12.0)	13 (2.0)	<0.001	12 (11.1)	9 (8.3)	0.491
**Coronary stent, n**	9 (7.2)	2 (0.3)	<0.001	4 (3.7)	2 (1.9)	0.683
**Preoperative LVEF, %**	57 [50–59]	59 [57–63]	<0.001	57 [52–59]	57 [53–61]	0.177
**Surgery type**						
**Revision, n**	5 (4.0)	34 (5.3)	0.553	4 (3.7)	2 (1.9)	0.683
**Coronary artery bypass graft, n**	28 (22.4)	26 (4.0)	<0.001	17 (15.7)	13 (12.0)	0.431
**Valvular heart surgery, n**	63 (50.4)	395 (61.2)	0.024	60 (55.6)	62 (57.4)	0.784
**Thoracic aortic surgery, n**	8 (6.4)	63 (9.8)	0.008	8 (7.4)	6 (5.6)	0.783
**Other cardiac surgery, n**	18 (14.4)	102 (15.8)	0.690	15 (13.9)	19 (17.6)	0.455
**Combined procedure, n**	8 (6.4)	59 (9.1)	0.319	8 (7.4)	8 (7.4)	0.999
**Medication history**						
**Angiotensin receptor blocker, n**	19 (15.2)	64 (9.9)	0.082	27 (14.3)	27 (14.3)	0.999
**Angiotensin converting enzyme inhibitor, n**	6 (4.8)	32 (5.0)	0.939	16 (14.8)	15 (13.9)	0.846
**Calcium channel blocker, n**	28 (22.4)	61 (9.5)	<0.001	25 (23.1)	26 (24.1)	0.873
**Beta-blocker, n**	29 (23.2)	85 (13.2)	0.004	23 (21.3)	24 (22.2)	0.869
**Statin, n**	28 (22.4)	54 (8.4)	<0.001	23 (21.3)	21 (19.4)	0.735
**Digitalis, n**	16 (12.8)	169 (26.2)	0.001	15 (13.9)	18 (16.7)	0.570
**Warfarin. n**	13 (10.4)	220 (34.1)	<0.001	13 (12.0)	8 (7.4)	0.251
**Furosemide, n**	34 (27.2)	191 (29.6)	0.587	30 (27.8)	28 (25.9)	0.759

Values are expressed as median (interquartile range) or number (%). ASA group = Aspirin administration before surgery for more than 7 days without discontinuation. No-ASA group = aspirin discontinuation before surgery for more than 7 days or did not take aspirin during the month before surgery. LVEF = left ventricular ejection fraction.

## Results

A total of 770 patients were analyzed after exclusion explained in the Methods (Fig 1). Among these patients, 399 patients (51.8%) developed AKI as defined by the KDIGO criteria with stage 1 (n = 275, 35.7%), stage 2 (n = 94, 12.2%) or stage 3 (n = 30, 3.9%) and 128 (16.6%) required hemodialysis within the first seven postoperative days.

**Fig 2:**
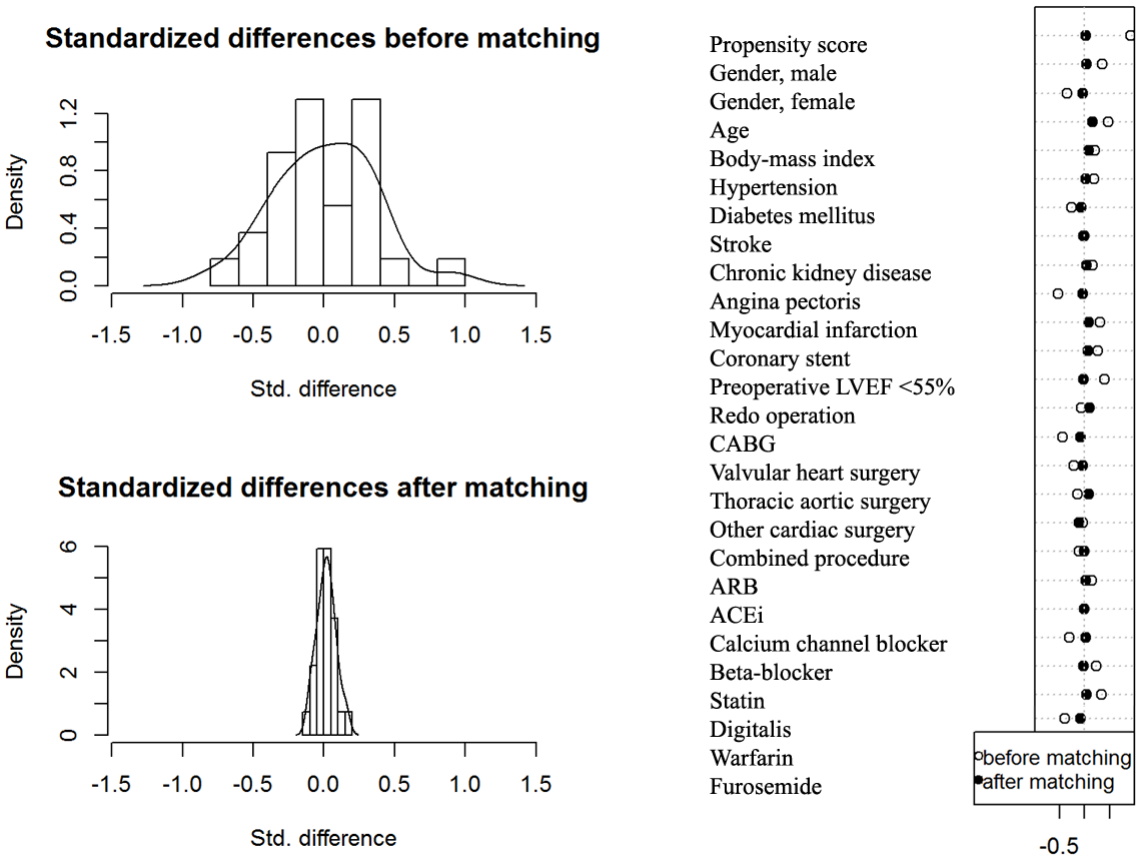
Histogram (left) and covariate balance plot (right) of distribution of standardized differences in the propensity scores between patients with aspirin use until surgery without discontinuation > 24 hours and patients who did not administer aspirin within one month before surgery or discontinue > 24 hours before and after matching. Values in the X-axis represent the percent standardized difference of covariates between the aspirin and non-aspirin groups. CABG = coronary artery bypass graft, ARB = angiotensin receptor blocker, ACEi = Angiotensin converting enzyme inhibitor.

Patient and surgical characteristics, baseline medical status and preoperative medications of the study population are presented in Table 1. Of the 950 patients initially reviewed, 125 (13.2%) had aspirin preoperatively without discontinuation more than 24 hours. 180 (18.9%) patients took aspirin preoperatively but stopped aspirin more than 24 hours but not longer than 7 days and the remaining 645 (67.9%) patients stopped aspirin for longer than 7 days or did not administer aspirin within 30 days before surgery. Most patients took aspirin 100 mg once daily (n = 195, 96.5%) and the remaining patients administered 75 mg once daily. The patients with aspirin were older, had more men, frequent history of chronic kidney disease, diabetes mellitus, coronary stent, poor cardiac systolic function and angina pectoris and underwent CABG surgery more frequently than the patients without preoperative aspirin. These patients also took preoperative calcium channel block, beta-blocker and statin more frequently and digitalis and warfarin less frequently than those without aspirin. The propensity score-matched group set comprised of 216 patients. As shown by the *P*-values of the Mann-Whitney *U* test and chi-square or Fisher exact test, the balance of both aspirin and non-aspirin groups was good for the parameters used for contributors to the propensity score (overall balance test: χ^2^ = 6.812, *P* = 1.000) (Table 1)(Fig 2).

Table 2 shows the results of both univariable and multivariable analyses of predictors for AKI within all KDIGO stages in both the total and matched sample. Multivariable logistic regression in entire cohort revealed that aspirin without discontinuation had marginally significant protective effect, but regression in the matched cohort showed aspirin’ s significant protective effect against postoperative AKI. (entire sample: odds ratio [OR] 0.46, 95% confidence interval [CI] 0.21–0.98, *P* = 0.048; after propensity score matching: OR 0.39, 95% CI 0.22–0.67, *P* = 0.001).

**Table 2:** Univariable and multivariable analysis of patient characteristics associated with acute kidney injury.

Variable	Univariable analysis in the entire cohort (n = 770)		Multivariable analysis in the entire cohort (n = 770)		Multivariable analysis in the matched cohort (n = 216)	
	Odds Ratio (95% CI)	*P*-value	Odds Ratio (95% CI)	*P*-value	Odds Ratio (95% CI)	*P*-value
**Age, years**	1.03 (1.02–1.05)	<0.001	1.04 (1.02–1.07)	0.001		
**History of chronic kidney disease, n**	3.39 (1.59–7.22)	0.002				
**Preoperative GFR, mL/min/1.73 m**^**2**^	0.99 (0.98–0.99)	<0.001				
**Preoperative hematocrit < 30%**	3.60 (1.98–6.52)	<0.001	4.94 (1.59–15.39)	0.006		
**Preoperative albumin < 4.0 mg/dl**	2.16 (1.55–3.02)	<0.001			5.22 (1.17–23.32)	0.030
**Preoperative uric acid > 5.5 mg/dl**	1.47 (1.04–2.08)	0.030	1.78 (0.98–3.23)	0.060		
**Preoperative serum creatinine > 1.2 mg/dl**	1.84 (1.25–2.69)	0.002				
**Preoperative left ventricular ejection fraction < 55%**	1.96 (1.34–2.88)	0.001			16.53 (3.16–86.52)	0.001
**Maintenance aspirin until surgery without discontinuation 24 hour or more**	0.59 (0.40–0.87)	0.007	0.46 (0.21–0.98)	0.048	0.39 (0.22–0.67)	0.001
**Preoperative furosemide medication, n**	2.19 (1.59–3.02)	<0.001	1.89 (1.05–3.38)	0.033		
**Operation time, every 1 hour**	1.28 (1.18–1.37)	<0.001				
**CPB time, every 1 hour**	1.43 (1.30–1.57)	<0.001	1.57 (1.33–1.85)	<0.001	1.01 (1.01–1.02)	0.002
**Intraoperative transfusion**						
**pRBC, unit**	1.10 (1.04–1.15)	<0.001				
**FFP, unit**	1.16 (1.09–1.23)	<0.001				
**Platelet concentrate, unit**	1.09 (1.04–1.14)	<0.001				
**Intraoperative mean arterial pressure < 60 mmHg**	2.57 (1.18–5.61)	0.018				
**Intraoperative epinephrine infusion, n**	2.43 (1.38–4.29)	0.002				
**Intraoperative norepinephrine infusion**	1.38 (1.04–1.84)	0.027				

CI = confidence interval; GFR = glomerular filtration rate, pRBC = packed red blood cells; FFP = fresh frozen plasma.

**Fig 3:**
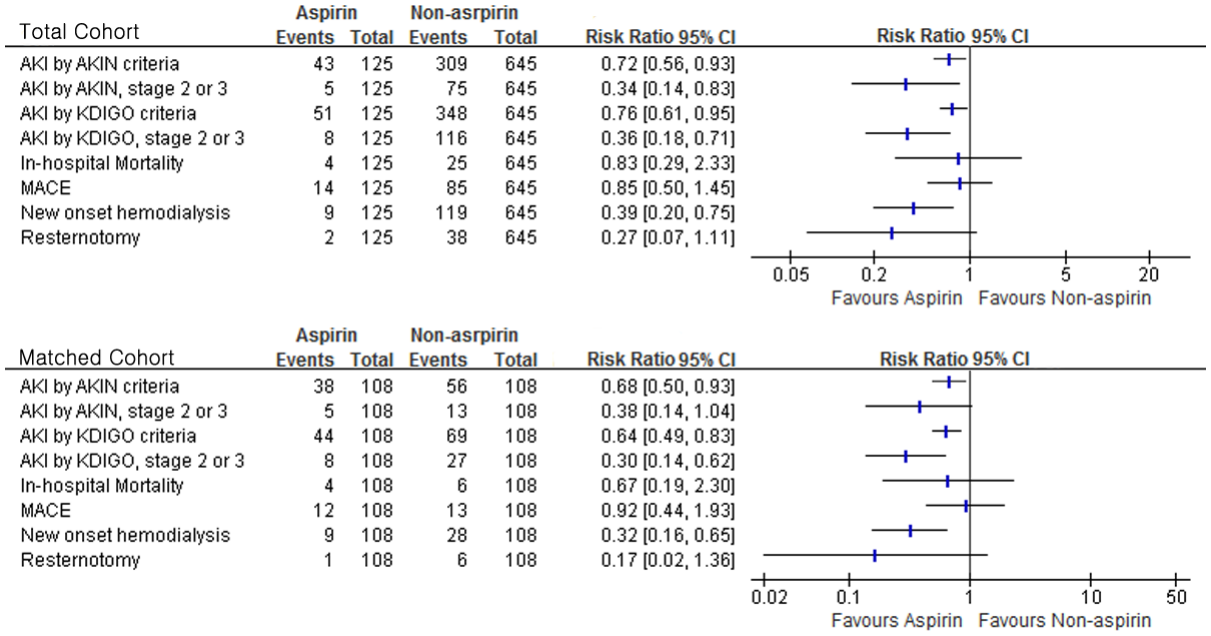
Comparison of risk ratios for clinical outcomes including acute kidney injury between patients with and without preoperative aspirin therapy. AKIN criteria = acute kidney injury network criteria; KDIGO criteria = Kidney Disease Improving Global Outcomes criteria; MACE = major adverse cardiovascular event; CI = confidence interval.

Table 3 and Fig 3 shows the comparison of the incidence of postoperative AKI and postoperative outcomes according to preoperative aspirin. Preoperative maintenance of aspirin was associated with less incidence of AKI defined by KDIGO both in the entire and matched cohort (n = 44 [40.7%] vs. 69 [63.9%] in aspirin and non-aspirin group, respectively in matched sample, relative risk (RR) 0.64, 95% CI 0.49–0.83, *P* = 0.001). This was also true in terms of AKI defined by AKIN criteria determined during the postoperative two days and was also true only for the second and third stages of both AKI criteria in both entire and matched sample. Preoperative aspirin was associated with decreased postoperative hospital stay after matching (12 [9–18] days vs. 16 [10–25] in aspirin and non-aspirin group, respectively, *P* = 0.038). Intraoperative estimated blood loss or blood loss during the surgery day calculated by difference in hematocrit showed no difference according to aspirin administration in both the entire and matched cohort (calculated blood loss: 2090 [1550–2820] vs. 2180 [1410–2950] in the aspirin and non-aspirin group, respectively after matching, *P* = 0.983).

**Table 3:** Intraoperative bleeding amount and postoperative outcomes in entire and matched cohort.

Characteristic	Entire cohort			Matched cohort		
	Aspirin group (n = 125)	Non-Aspirin group (n = 645)	*P*-value	Aspirin group (n = 108)	Non-Aspirin group (n = 108)	*P*-value
**Intraoperative estimated blood loss, ml**	1100 [800–2000]	1100 [700–2000]	0.896	1100 [800–2075]	1000 [700–2000]	0.587
**Calculated blood loss during the surgery day, ml**	2080 [1490–2820]	2010 [1400–2800]	0.232	2090 [1550–2820]	2180 [1410–2950]	0.983
**Intraoperative transfusion requirements**						
**Red blood cells, n**	2 [1–5]	2 [0–4]	0.282	2 [1–54]	1 [0–5]	0.336
**Fresh frozen plasma, n**	3 [0–3]	3 [0–4]	0.182	3 [0–3]	2 [0–3]	0.940
**Platelets, n**	0 [0–4]	0 [0–4]	0.450	0 [0–4]	0 [0–3]	0.837
**Hospital stay, total, days**	17 [14–7]	19 [14–29]	0.317	17 [14–25]	19 [13–31]	0.631
**Hospital stay, postoperative, days**	12 [9–19]	16 [9–25]	0.009	12 [9–18]	16 [10–25]	0.038
**ICU stay, days**	2 [1–5]	3 [2–5]	0.012	2 [1–5]	3 [2–5]	0.265
**IABP insertion during and after surgery, n**	10 (7.8)	38 (5.9)	0.372	7 (6.5)	8 (7.4)	0.789
**ECMO insertion during and after operation, n**	2 (1.6)	15 (2.3)	0.999	2 (1.9)	3 (2.8)	0.999
**Atrial fibrillation during postoperative three days, n**	5 (4.0)	58 (9.0)	0.062	4 (3.7)	9 (8.3)	0.153

Values are expressed as mean (SD), median [interquartile ranges] or number (%). The incidences of complication were investigated during postoperative hospital stay. ICU = intensive care unit; AKI = acute kidney injury; IABP = intra-aortic balloon pump; ECMO = extracorporeal membrane oxygenation.

## Discussion

In this propensity-scored matched retrospective observational study, we demonstrated that preoperative low dose aspirin administration without discontinuation was associated with decreased postoperative AKI incidence in terms of both KDIGO and AKIN criteria and decreased requirements for hemodialysis. Bleeding amounts and transfusion requirements were not different according to preoperative aspirin in both entire and matched sample. Preoperative aspirin administration was associated with shortened postoperative hospital stay. These results implicate that maintenance of aspirin before cardiac surgery may decrease the development of postoperative AKI and hemodialysis requirements without increasing bleeding risk.

Previous guidelines in the early 2000s on the use of aspirin prior to cardiac surgery recommended to stop aspirin for 2 to 10 days preoperatively due to the concern regarding increased perioperative bleeding [35]. However, following studies on the association between preoperative aspirin and the clinical outcomes after cardiac surgery reported potential benefits on postoperative major morbidity and mortality [7–10]. The most recent American College of Cardiology Foundation (ACCF)/AHA guidelines for CABG surgery in 2011 recommended that aspirin of 100 mg to 325 mg once daily should be administered to CABG patients preoperatively, although level of evidence is still low [36].

Postoperative administration of aspirin improves graft patency in CABG surgery, decreases ischemic complications, and improves postoperative clinical outcomes [37–40]. In 2002, a large prospective trial by Mangano et al.[38] showed that early use of aspirin after CABG surgery was associated with a reduced risk of death and ischemic complications of major organs. 2016 ACC/AHA Guidelines Focused Update have discussed on the dual antiplatelet therapy (DAPT) in patients undergoing CABG and addressed the benefits of DAPT in off-pump CABG patients in terms of improved graft patency and clinical outcomes [41].

Although increasing number of patients are taking aspirin perioperatively, reliable evidences are still lacking whether aspirin should be continued until the surgery or discontinued preoperatively [4–6]. High quality studies on the association between preoperative aspirin and the outcomes of cardiac surgery were relatively rare and the results so far were not consistent [7–10,13–15] In a previous observational study of 2868 patients, preoperative aspirin administration was associated with decreased postoperative morbidity including major cardiovascular and cerebral complications, renal failure, length of ICU and hospital stay and short-term mortality [9]. In another retrospective cohort study, those who administered aspirin within 24 hours before CABG surgery were associated with decreased early postoperative mortality without increasing risk of reoperation for bleeding [10]. However, a study reviewing 4143 patients undergoing CABG from cardiovascular information registry found late discontinuation of aspirin resulted in no difference in postoperative composite outcomes and was associated with increased bleeding complications [13]. A meta-analysis reviewing eight randomized controlled trials concluded that preoperative aspirin increases postoperative bleeding without significant reduction in mortality [5]. However, subgroup analysis showed that bleeding depends on the dose of aspirin and can be avoided by using preoperative aspirin less than 325 mg/day.

There are several possible explanations as to the association between preoperative aspirin and postoperative AKI. Firstly, aspirin may improve microcirculatory environment in the kidney by suppressing thrombogenic condition produced by surgical stress. Aspirin’s ability to inhibit platelet cyclooxygenase-1, thereby blocking production of thromboxane A_2_ has been regarded to mediate the benefit of aspirin to prevent ischemic events [42]. Impaired renal perfusion due to decreased microvascular flow may contribute to the renal ischemic injury during cardiac surgery with frequent low cardiac output and cardiogenic shock. Renal ischemia-reperfusion injury was considered to be one of the etiology of AKI development [17,18]. Secondly, aspirin may decrease the incidence of AKI by its anti-inflammatory action [19,20]. Inflammation following ischemia-reperfusion injury has been designated as a main pathophysiology of AKI [20]. This was suggested from the observations that anti-inflammatory strategies have shown some effects in mitigating AKI incidence, although evidence was not strong.

The other risk factors for cardiac surgery-associated AKI revealed by our study are mostly consistent with previous studies. Previous studies have reported that old age, preoperative anemia, high uric acid levels, low serum albumin levels, furosemide medication, long surgery and CPB time, intraoperative inotropes use and large volume transfusion were associated with postoperative AKI [21,43,44]. Preoperative renal dysfunction was associated with postoperative AKI [21]. When we analyzed the association between preoperative glomerular filtration rate (GFR) in chronic kidney disease patients and postoperative AKI, higher stages of AKI defined by KDIGO criteria were associated with the higher stages of chronic renal failure (S2 Table) [45].

There are several limitations that needed to be addressed. Firstly, we attempted to remove any potential confounders to influence the endpoints of our study using propensity score matching. Extensive matching of patient demographics, baseline cardiac history, surgery types and other medications was performed and the results showed well balancing of matched variables. However, due to the retrospective and single-center design, unknown bias not used in the matching may still confound our results and external validity is limited. Secondly, the relationship between preoperative aspirin and postoperative AKI is only an association. Any causal relationship or mechanisms that aspirin can decrease postoperative AKI cannot be suggested from our study results. Thirdly, data are lacking regarding optimal timing and dosing of aspirin. The dose used in our population was very homogeneous as a low dose (100 mg in 96.5% patients) and we used the cutoff of preoperative 24 hours for aspirin discontinuation. Therefore, although we cannot provide the effect of dose and timing of discontinuation of aspirin on the postoperative AKI and clinical outcomes, the effect of low dose aspirin could be evaluated due to the absence of heterogeneous dose spectrum. Fourthly, the impact of postoperative aspirin administration was not investigated or adjusted in our analysis. Standard protocol of our hospital was to begin aspirin within 1 day after CABG and to restore aspirin medication 1 day after surgery in patients who administered aspirin preoperatively if there is no evidence of significant postoperative bleeding. As we matched the surgery type, the postoperative aspirin medication would be the same between the two groups in the matched sample.

In conclusion, preoperative low dose aspirin administration was associated with decreased postoperative AKI in terms of both KDIGO and AKIN criteria, decreased hemodialysis and shortened postoperative hospital stay. However, aspirin use was not associated with low in-hospital mortality. Perioperative bleeding and transfusion requirements did not differ after propensity score matching for demographic, cardiac status, surgical types and other medications. From these results, we can conclude that maintenance of low dose aspirin before cardiac surgery may decrease postoperative AKI and hemodialysis requirements without increasing bleeding. However, due to the limitation of retrospective study, a large randomized studies are required to determine conclusively the safety and effectiveness of preoperative aspirin in setting of cardiac surgery.

## Supporting information

S1 Checklist A STROBE checklist for the present study. (DOC)

S1 Table Logistic regression analysis of three aspirin groups. (DOCX)

S2 Table Subgroup analysis of the patients with chronic kidney disease. (DOCX)

S1 File A dataset for the present study. (XLSX)
