# Supplementary material for: Preoperative aspirin use and acute kidney injury after cardiac surgery: A propensity-score matched observational study
Source: PLoS One. 2017 May 4;12(5):e0177201. doi: 10.1371/journal.pone.0177201 (PMC5417712; doi:10.1371/journal.pone.0177201)
Supplement: S1 Table — (DOCX) [file pone.0177201.s002.docx]

Table S1. Multivariable analysis including three aspirin groups.

|  | **Multivariable analysis** |  |
| --- | --- | --- |
| **Variable** | **Odds ratio (95% CI)** | ***P*-value** |
| **Age, years** | 1.05 (1.03 – 1.08) | <0.001 |
| **Preoperative hematocrit <30%** | 2.91 (1.18 – 7.19) | 0.021 |
| **Preoperative uric acid <5.5 mg/dL** | 1.94 (1.13 – 3.33) | 0.017 |
| **Preoperative furosemide** | 1.55 (0.92 – 2.62) | 0.098 |
| **ASA group 1 vs. group 3**  **ASA group 2 vs. group 3**  **ASA group 3** | 0.47 (0.22 – 0.88)  1.39 (0.76 – 2.53)  baseline | 0.036  0.281 |
| **CPB time, min** | 1.005 (1.003 – 1.008) | <0.001 |
| **Intraoperative epinephrine** | 2.18 (0.91 – 5.22) | 0.082 |

This analysis was performed in 950 patients (after exclusion of abdominal aortic surgery and off-pump CABG). Patients were divided into three groups according to aspirin intake pattern as follows.

Group 1 (n=125): patients who took aspirin until the surgery without discontinuation of aspirin for more than 24 hours; Group 2 (n=180): patients who took aspirin before surgery, but stopped for more than 24 hours but less than 7 days. (i.e. patients who might have the residual effects of aspirin); Group 3 (n=645): patients who stopped aspirin for more than 7 days or who never administered aspirin during the one month before surgery.

CI = confidence interval.

The results of univariable analysis of aspirin three groups and postoperative acute kidney injury are as follows.

Group 1 vs. Group 3: Odds ratio = 0.50, 95% CI = 0.36 to 0.71, *P*<0.001

Group 2 vs. Group 3: Odds ratio = 1.21, 95% CI = 0.89 to 1.67, *P*=0.212

Group 3 (baseline)
