# Supplementary material for: Preoperative aspirin use and acute kidney injury after cardiac surgery: A propensity-score matched observational study
Source: PLoS One. 2017 May 4;12(5):e0177201. doi: 10.1371/journal.pone.0177201 (PMC5417712; doi:10.1371/journal.pone.0177201)
Supplement: S2 Table — (DOCX) [file pone.0177201.s003.docx]

Table S2. Subgroup analysis of the patients with chronic kidney disease (n = 40)

|  | **Patients (n)** | **Postoperative AKI according to KDIGO criteria (n, %)** | **Postoperative AKI, KDIGO stage 1/2/3 (n, %)** |
| --- | --- | --- | --- |
| **Stage 1** | 0 | 0 | 0 |
| **Stage 2** | 2 | 2 (100%) | 1 (50.0) / 1 (50.0) / 0 |
| **Stage 3a** | 7 | 5 (71.4%) | 3 (42.9) / 2 (28.6) / 0 |
| **Stage 3b** | 13 | 12 (92.3%) | 4 (30.8)/ 6 (46.2) / 2 (15.4) |
| **Stage 4** | 8 | 5 (62.5%) | 3 (37.5) / 0 / 2 (25.0) |
| **Stage 5** | 10 | 7 (70%) | 1 (10.0) / 0 / 6 (60.0) |
| **Total** | 40 | 31 (77.5%) | 12 (30.0) / 9 (22.5) / 10 (25.0) |
| ***P*-value*** |  | 0.445 | 0.055 |

Patients with chronic kidney disease (CKD) among 770 patients were analyzed.

*P*-values are the results of chi-square test.

The stages of CKD were defined as follows.

Stage 1: Kidney damage with normal or increased glomerular filtration rate (GFR, >90 mL/min/1.73 m^2^)

Stage 2: Mild reduction in GFR (60-89 mL/min/1.73 m^2^)

Stage 3a: Moderate reduction in GFR (45-59 mL/min/1.73 m^2^)

Stage 3b: Moderate reduction in GFR (30-44 mL/min/1.73 m^2^)

Stage 4: Severe reduction in GFR (15-29 mL/min/1.73 m^2^)

Stage 5: Kidney failure (GFR <15 mL/min/1.73 m^2^ or dialysis)

In stage 1 and stage 2 CKD, the presence of one or more of the following markers of kidney damage are required to establish the diagnosis.

1. Albuminuria (albumin excretion >30 mg/24 hr or albumin:creatinine ratio >30 mg/g [>3 mg/mmol])

2. Urine sediment abnormalities

3. Electrolyte and other abnormalities due to tubular disorders

4. Histologic abnormalities

5. Structural abnormalities detected by imaging

6. History of kidney transplantation in such cases

When we analyzed the association between preoperative GFR of CKD patients as a continuous variable with the postoperative AKI, the association was not significant.

Univariable analysis, odds ratio 1.015, 95% confidence interval, 0.97 to 1.07, *P* =0.540.
